# Similar Outcomes of Web-Based and Face-to-Face Training of the GRADE Approach for the Certainty of Evidence: Randomized Controlled Trial

**DOI:** 10.2196/43928

**Published:** 2023-06-06

**Authors:** Ružica Tokalić, Tina Poklepović Peričić, Ana Marušić

**Affiliations:** 1 Department of Research in Biomedicine and Health Center for Evidence-based Medicine University of Split School of Medicine Split Croatia; 2 Cochrane Croatia University of Split School of Medicine Split Croatia

**Keywords:** Grading of Recommendations Assessment, Development and Evaluation, GRADE, education, online, face-to-face, evidence-based medicine, guideline, randomized controlled trial, RCT, randomized, evidence, assessment, teaching, medical education, research method, online education, library science, information science, medical librarian

## Abstract

**Background:**

The GRADE (Grading of Recommendations Assessment, Development and Evaluation) approach is a system for transparent evaluation of the certainty of evidence used in clinical practice guidelines and systematic reviews. GRADE is a key part of evidence-based medicine (EBM) training of health care professionals.

**Objective:**

This study aimed to compare web-based and face-to-face methods of teaching the GRADE approach for evidence assessment.

**Methods:**

A randomized controlled trial was conducted on 2 delivery modes of GRADE education integrated into a course on research methodology and EBM with third-year medical students. Education was based on the Cochrane Interactive Learning “Interpreting the findings” module, which had a duration of 90 minutes. The web-based group received the web-based asynchronous training, whereas the face-to-face group had an in-person seminar with a lecturer. The main outcome measure was the score on a 5-question test that assessed confidence interval interpretation and overall certainty of evidence, among others. Secondary outcomes included writing a recommendation for practice and course satisfaction.

**Results:**

In all, 50 participants received the web-based intervention, and 47 participants received the face-to-face intervention. The groups did not differ in the overall scores for the Cochrane Interactive Learning test, with a median of 2 (95% CI 1.0-2.0) correct answers for the web-based group and 2 (95% CI 1.3-3.0) correct answers for the face-to-face group. Both groups gave the most correct answers to the question about rating a body of evidence (35/50, 70% and 24/47, 51% for the web-based and face-to-face group, respectively). The face-to-face group better answered the question about the overall certainty of evidence question. The understanding of the Summary of Findings table did not differ significantly between the groups, with a median of 3 correct answers to 4 questions for both groups (*P*=.352). The writing style for the recommendations for practice also did not differ between the 2 groups. Students’ recommendations mostly reflected the strengths of the recommendations and focused on the target population, but they used passive words and rarely mentioned the setting for the recommendation. The language of the recommendations was mostly patient centered. Course satisfaction was high in both groups.

**Conclusions:**

Training in the GRADE approach could be equally effective when delivered asynchronously on the web or face-to-face.

**Trial Registration:**

Open Science Framework akpq7; https://osf.io/akpq7/

## Introduction

### Background

The extent of confidence in the desirable effects of an intervention outweighing the undesirable ones is a valuable indicator in the strength of recommendations for clinical practice [1]. The GRADE (Grading of Recommendations Assessment, Development and Evaluation) approach is a system that has been designed by a group of international guideline developers for transparent evaluation of the certainty of evidence and the development of transparent, robust, and trustworthy guidelines. The process of GRADE-ing should begin with a formulated health care question in a patient, population, or problem; intervention; comparison; and outcome format that is then used to systematically search relevant databases and select relevant studies. Patient-relevant outcomes are then assessed for each included study while focusing on study limitations, precision, the directness of evidence, the consistency of results, and possible confounders, among others. The certainty of evidence is then decided for each outcome, ranging from very low to high. The overall quality of evidence for the main health care question will depend on the lowest-rated critical outcome. To decide on the final recommendation, guideline developers use the overall certainty of the evidence, balance of unwanted effects and values, and preferences of the patients [2,3].

GRADE is not the first nor the only approach for assessing the certainty of evidence and assigning the strength of recommendations. It is, however, the approach used in Cochrane systematic reviews and many clinical practice guidelines. The GRADE approach includes a Summary of Findings (SoF) table to make the process of judging evidence and translating it into a recommendation more accessible for a broader audience, primarily for end users—clinicians, patients, and policy makers. It is a systematic, transparent, and concise report of key information that includes the certainty of evidence and the effect size of an intervention used for each outcome and across outcomes [3]. SoF tables in systematic reviews ease the understanding of the certainty of evidence and the review’s key points [4].

To effectively apply clinical practice guidelines and other summarized formats of evidence, health care providers need to have the evidence-based medicine (EBM) skills necessary for understanding and the application of clinical practice guidelines. Aside from occasional specialized courses and a short video series [5], there are no official GRADE educational resources. Research methodology and statistics courses in medical schools provide the basis necessary for understanding GRADE [6].

Web-based education uses web-based technologies for knowledge and skills improvement. It can be asynchronous, in which users can individually access it anytime and progress through it at their own pace. It can also be synchronous, in which users have to access it at certain times, usually in some form of webinars. Web-based educational interventions have shown noninferior results in learning and participant satisfaction outcomes compared to face-to-face learning in medicine, including communication skills and cardiology [7,8]. Asynchronous web-based education was successfully used as supplementary learning in emergency medicine and for knowledge on systematic reviews [9,10]. In addition, for EBM, the cost-effectiveness of web-based education was superior to that of traditional face-to-face learning [11]. In this study, we wanted to test if the addition of a GRADE-focused educational content into a basic EBM course could increase the understanding of SoF tables among medical students and whether the delivery mode of that content influences the learning outcomes. We also assessed how the 2 modes of GRADE training affected the application of the GRADE approach in providing recommendations for clinical practice.

### Aim

The aim of this study was to determine the effectiveness of a web-based educational intervention for the GRADE approach to evidence assessment, compared to traditional classroom education, in terms of knowledge and the understanding of the SoF table.

## Methods

### Trial Design and Participants

This was a parallel-group randomized controlled trial. Participants were third-year medical students in Croatian- and English-language programs at the University of Split School of Medicine. Students were attending a mandatory course on research methodology and EBM, described in a previously published study [6]. To be a part of the study, they had to be 18 years or older and fluent in English, both of which are a part of our School of Medicine requirements for enrollment. All students had to pass 2 previous courses (in the first and second years of their medical studies, respectively) to attend the third-year course and had a similar level of knowledge about research methodology and EBM [6,12].

### Intervention

The web-based educational intervention was based on the Cochrane Interactive Learning (CIL) module 7, titled “Interpreting the findings” [13]. This educational module is completely on the web and asynchronous. The duration of the module is 90 minutes, and it covers the interpretation of statistical results, risk of bias, and interpretation of levels of evidence using the GRADE approach. The intervention group got the access to the web-based module in the classroom at the same time as the control group. Participants used the faculty-provided electronic devices to access the module. The control group had a traditional face-to-face seminar in the classroom, which was taught by a lecturer with knowledge and experience in GRADE and is a Cochrane systematic review author but had no involvement in the CIL module development. The presentations for the face-to-face seminar had the exact same content as the web-based module, equal in graphical design and duration.

After 90 minutes, the participants took the same test, hosted on the SurveyMonkey platform (SurveyMonkey Inc) [14]. There were no time limitations for the test, that is, the participants could spend as much time as they needed to complete the test. During the test, one of the researchers was present in the classroom of each trial group.

The first part of the SurveyMonkey test was a brief sociodemographic questionnaire, which included questions on participants age, gender, level of education, current research activities, and authorship of research publications. The participants were also asked to assess their knowledge of the GRADE approach (ranging from 1=little to none to 5=excellent), as well as their familiarity with Cochrane and systematic reviews (from 1=not at all to 5=extremely familiar).

After that, the participants took a test that assessed their knowledge of the GRADE approach. Five multiple-choice questions on statistical terms and their evaluation were taken from the official assessment for the CIL module [13]. The questions covered the topics of confidence interval interpretation using a forest plot, the expression of standardized mean difference, funnel plot interpretation, the assessment of overall certainty of evidence, and the rating of a body of evidence. Some of the questions had only one correct answer, whereas some had multiple correct statements. For those questions, points were awarded only if the participants selected all of the correct statements.

The final part of the test evaluated the participants’ understanding of an SoF table, which was evaluated with 4 open-ended questions linked to an SoF example [15]. The participants were asked to (1) give a recommendation for clinical practice based on the SoF table information, (2) determine target patient groups and possible exceptions or exclusion criteria, (3) find sample sizes for specific outcome analyses, and (4) find reasons for grading some of the evidence as very low certainty.

The SoF table and the questions from the test are available in Multimedia Appendix 1 [15].

### Outcomes

The primary outcome for this study was the knowledge measured by the 5 questions from the formal CIL module. The knowledge was measured in 2 ways: as the overall scores for the test and the number of students correctly answering each question.

There were 3 secondary outcomes:

1. The understanding of the SoF table was measured by 4 questions related to an SoF table example. The results were expressed as the number and percentage of students with correct answer to individual questions and the total score for the whole group.
2. Participants’ satisfaction with and opinion about the course was measured using 10 questions with Likert-type statements, with scoring ranging from 1=I do not agree at all to 7=I fully agree.
3. The style of writing of a recommendation for clinical practice in the answer to the first of the 4 questions about the SoF table: we assessed the style according to the National Institute for Health and Care Excellence (NICE) instructions for writing recommendations [16]. These instructions advise that recommendations focus on the action and procedure, reflect the strength of the recommendation, and have clear and precise patient-oriented language. The 3 main categories from the NICE writing recommendations were used:
  The *focus of the action* was assessed according to three elements from the guidebook: (1) the verb use, (2) target population, and (3) context or setting for the recommendation. Each element was graded as 1 if the element was present in the text or 0 if it was not present.
  The opinion of the students about the *strength of recommendation* was graded as 1 or 0, based on the use of verbs for 3 levels of recommendations: “must” or “must not” for interventions that must be considered, “should” or “should not be offered” for interventions that should be considered, and “could offer” or “consider” for interventions that could be considered in clinical practice.
  *Patient-centered language* assessment was guided by the guidebook recommendation to use verbs such as “offer,” “discuss,” and “consider,” instead of “give” and “prescribe.” Responses for this outcome were grouped into three categories: (1) offer (including “offer,” “consider,” “suggest,” “recommend,” “advise,” and “could help”); (2) give and prescribe (including “(do not) give,” “(do not) prescribe,” “supplement with,” and passive voice); and (3) no recommendation (response included no elements of clinical decision-making).

Two independent assessors (RT and TPP) rated all of the responses. Inconsistencies in their ratings were resolved with the help of a third author. κ statistics were used to determine the level of agreement for each of the 3 categories.

### Sample Size

Based on the primary outcome and the assumption that there would be no significant differences between groups, we calculated the minimal sample size using a web-based calculator [17]. The allocation ratio was set to 1:1, the α value was .05, and the power was 0.8. We calculated that we needed 16 participants per group to obtain a 10% difference (out of a maximum of 11 correct statements). We hypothesized that there would be a lesser, nonsignificant difference than that in our previous studies, as we did not expect the groups to differ [6,16]. With the predicted attrition rate of 10%, we aimed at 36 participants in total.

### Randomization and Masking

We used a simple randomization method [18]. One author prepared the list of the participants and randomized them into 2 groups. The group allocation was posted on the web the day before the intervention took place. Participants did not know beforehand which group would attend the web-based course. Web-based group participants got access to the web-based content (access usernames and passwords) at the very beginning of the intervention. Because of the nature of the intervention, it was not possible to completely mask the participants. The groups got the same treatment, setting, and measurements, aside from the intervention. As we could not control for individual students’ study time with regard to EBM and GRADE, we included the questions on self-assessed knowledge of the GRADE approach and familiarity with Cochrane collaboration in the demographic part of the test. Data analysis was masked: the researchers who analyzed the responses and compared the groups were not aware of group allocation.

### Statistical Methods

Sociodemographic characteristics of participants are presented as absolute numbers and percentages. Group results are presented as medians and 95% Cis. The distribution of results was tested using the Kolmogorov-Smirnov test, and group results were compared using the Mann-Whitney *U* test. The results for separate questions and recommendations were presented as absolute values and percentages of correct answers and compared between groups using the Fischer exact test. To address multiple comparison bias, we performed a sequential Holm-Bonferroni adjustment. Analysis was conducted using MedCalc Statistical Software (version 16.4.3; MedCalc Software bvba) [19] and JASP software (version 0.8.6; JASP Team).

### Ethics Approval

The study was approved by the Ethics Committee of the University of Split School of Medicine (class 003-08/19-03/0003; registration 2181-198-03-04-19-0044). The participants gave informed consent, and the data were kept according to the General Data Protection Regulation.

## Results

**Figure 1:**
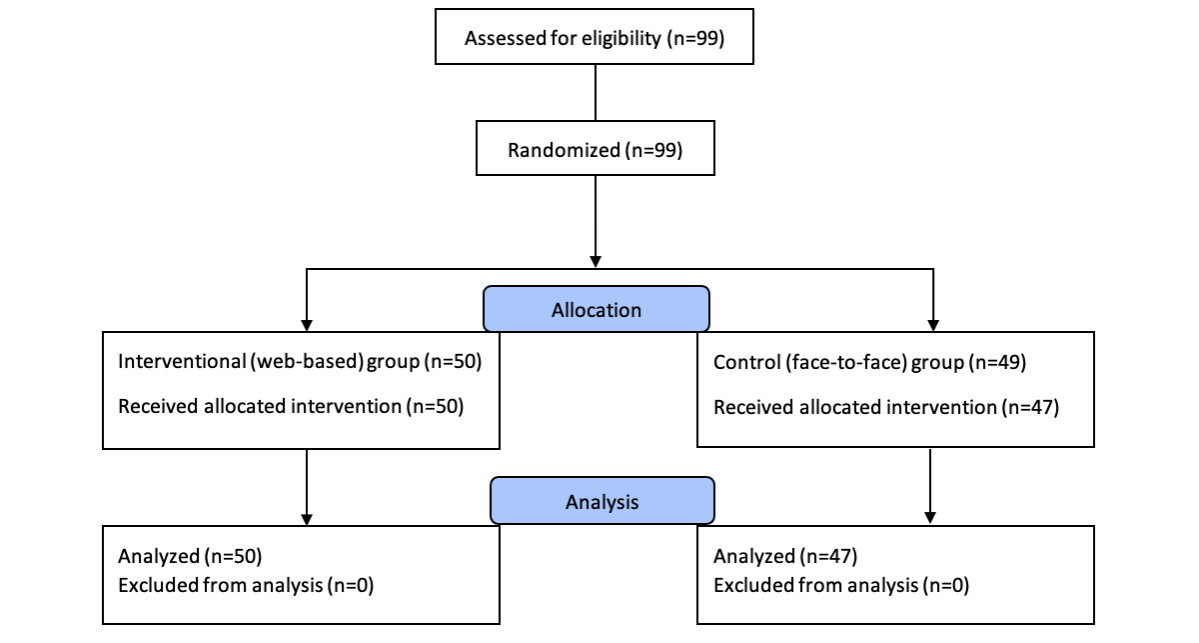
Flow diagram of the participants in the study.

The participant flow diagram is shown in Figure 1. In all, 50 participants received the web-based intervention, and 47 participants received the face-to-face intervention. Two participants did not attend the allocated intervention due to personal reasons. The median age was 21 (IQR 21-23) years. The groups did not differ in their current research experience or self-assessed GRADE knowledge (Table 1). Satisfaction with the intervention was high in both groups, and both groups reported that they will apply the knowledge in their work and learn more about interpreting and grading the quality of evidence. (Table 2).

**Table 1:** Demographic data, previous research experience, and self-assessed knowledge of the GRADE^a^ approach^b^.

Item	Web-based training (n=50)	Face-to-face training (n=47)
Gender (female; total: n=90, web-based training: n=48, face-to-face training: n=42), n (%)	29 (60)	27 (64)
Age (years; total: n=89, web-based training: n=47, face-to-face training: n=42), median (IQR)	21.0 (21-23)	21.0 (21-23)
Level of completed education (high school; total: n=89, web-based training: n=47, face-to-face training: n=42), n (%)	45 (96)	40 (95)
Are you currently involved in research activities? (yes; total: n=85, web-based training: n=48, face-to-face training: n=37), n (%)	1 (2)	0 (0)
Authorship of a research publication in the last 5 years (yes; total: n=80, web-based training: n=47, face-to-face training: n=33), n (%)	1 (2)	1 (3)
Authorship of a systematic review (yes; total: n=88, web-based training: n=47, face-to-face training: n=41), n (%)	1 (2)	0 (0)
Authorship of a clinical practice guideline (yes; total: n=89, web-based training: n=48, face-to-face training: n=41), n (%)	0 (0)	0 (0)
How familiar are you with Cochrane collaboration? (1=not at all, 5=extremely familiar; n=89), median (95% CI)	2.0 (2-2)	2.0 (2-2)
How would you grade your knowledge of GRADE approach? (1=very low, 5=very high; n=88), median (95% CI)	2.0 (2-2)	2.0 (2-2)

^a^GRADE: Grading of Recommendations Assessment, Development and Evaluation.

^b^The numbers in parentheses indicate the number of responses in the questionnaire.

**Table 2:** Participants’ satisfaction with the training session, presented as median scores with 95% CI^a^.

Item	Web-based training (n=50), median (95% CI)	Face-to-face training (n=47), median (95% CI)
Overall, I am satisfied with the course (n=85)	5 (5-5)	5 (5-6)
This course was really useful (n=87)	5 (4-5)	5 (5-5.8)
This is a good way for learning GRADE^b^ approach for quality of evidence (n=87)	5 (4-5)	5 (4-5)
This course helped me to better understand the concepts related to GRADE (n=87)	5 (4-5)	4 (4-5)
The course covered too much content in a short period of time (n=88)	4 (4-5)	4 (3.2-5)
I think there was sufficient amount of interaction during this course (n=86)	5 (5-6)	5 (4-6)
I would recommend this course to my colleagues (n=85)	5 (4-5.1)	5 (4-5.1)
I did not find this course useful (n=84)	2 (2-4)	2 (2-3)
In future, I will apply what I learned at this course in my work and research (n=85)	5 (5-5)	5 (5-5)
In future, I will learn more about interpreting and grading the quality of evidence (n=84)	5 (4-6)	5 (4.8-5.2)

^a^The numbers in parentheses indicate the number of responses in the questionnaire.

^b^GRADE: Grading of Recommendations Assessment, Development and Evaluation.

The groups did not differ in the overall scores for the CIL test (*P*=.251, Mann-Whitney *U* test; Table 3). The median number of correct answers for both groups was 2 out of 4 (Table 3). Most students from both groups gave correct answers on the question about rating the body of evidence (35/50, 70% and 24/47, 51% of students from web-based and face-to-face training groups, respectively). The only difference between the 2 groups was in their answer to the question about the overall certainty of evidence, where the face-to-face group had significantly more correct answers then the web-based training group (*P*=.008, Fischer exact test; Table 3).

Understanding of the SoF table also did not differ significantly between the groups (median of 3 correct questions to 4 questions for both groups; *P*=.352, Fischer exact test; Table 4). Most correct answers in both groups were to questions about the number of participants in specific trials and the reason for low grading of the quality of evidence for some patient groups (Table 4).

For the analysis of how students phrased their recommendation for practice (Table 5), the κ score for the 2 assessors was for 0.68 for the patient-centered language, 0.88 for the strength of recommendation, and 1.0 for the focus of the action category. The students in both groups rarely used active verbs in writing their recommendations, and there was no difference between the groups (*P*=.118). The majority of them did not specify the setting for the recommendation in both groups (*P*=.151). Very few students in both groups (*P*=.151) addressed the setting (time or context) of their recommendation for practice. Both groups (*P*=.683) also wrote in a more patient-centered language, using verbs such as “offer,” “discuss,” and “consider.”

**Table 3:** Number (%) of correct answers and overall score for the 5 questions of the Cochrane Interactive Learning test.

Item	Web-based training (n=50)	Face-to-face training (n=47)	*P* value^a^
Understanding of confidence intervals in the interpretation of results of meta-analysis, n (%)	18 (36)	16 (34)	>.99
Identify ways of re-expressing the standardized mean difference, n (%)	14 (28)	25 (53)	.014
Interpret a funnel plot asymmetry, n (%)	9 (18)	12 (26)	.461
Determine the overall certainty of the evidence, n (%)	15 (30)	27 (57)	.008
Decide on rating up a body of evidence, n (%)	35 (70)	24 (51)	.064
Overall score, median (95% CI)	2 (1.0-2.0)	2 (1.3-3.0)	.251^b^

^a^Fischer exact test. *P* value for significance was set to .010 after Holm-Bonferroni adjustment.

^b^Mann-Whitney *U* test.

**Table 4:** Number (%) of correct answers and overall score on 4 test questions related to the Summary of Findings table.

Item	Web-based training (n=50)	Face-to-face training (n=47)	*P* value^a^
Based on this information, how would you formulate a recommendation for clinical practice? (would recommend; total: n=83, web-based training: n=48, face-to-face training: n=35), n (%)	27 (56)	15 (43)	.270
Would you consider any subgroups of patients, and if so, how?, n (%)	24 (48)	22 (47)	>.99
How many participants were there in trials that assessed death as an outcome?, n (%)	39 (78)	30 (64)	.178
Why was the quality of evidence for hospitalized children graded as very low?, n (%)	31 (62)	39 (83)	.025
Overall score, median (95% CI)	3 (2-3)	2 (2-2)	.35^b^

^a^Fischer exact test. *P* value for significance was set to .0125 after Holm-Bonferroni adjustment.

^b^Mann-Whitney *U* test.

**Table 5:** Number (%) of students who used specific writing style in their clinical practice recommendation based on the Summary of Findings table in the test^a^.

Category of writing recommendation and element		Web-based training (n=50), n (%)	Face-to-face training (n=47), n (%)	*P* value^b^
**Focus of the action**				
	Active verb	4 (8)	0 (0)	.118
	Target population	24 (48)	10 (21)	.010
	Setting (time or context)	2 (4)	6 (13)	.151
**Reflects the strength of recommendation**		28 (56)	32 (68)	.296
**Patient-centered language**				
	Offer	31 (62)	27 (57)	.683
	Give and prescribe	10 (20)	1 (2)	.008
	No recommendation	9 (18)	19 (40)	.024

^a^Students’ recommendations written in the answer to the first Summary of Findings question were assessed according to the presence of categories from the National Institute for Health and Care Excellence (NICE) writing recommendations. The number of categories was greater than the number of students as their recommendation could include more than one category element of the writing style.

^b^Fischer exact test. *P* value for significance was set to .007 after Holm-Bonferroni adjustment.

## Discussion

### Principal Findings

Our study showed that web-based education about GRADE methodology may not be different to face-to-face education, as measured by the CIL module overall test results. The face-to-face group was better at assessing evidence using the risk of bias. There were no differences between the groups in the overall understanding of the SoF table. Both groups had high levels of satisfaction with the intervention. These results should be evaluated in the context of additional educational resources in EBM courses for medical school students and taking into account a limited sample size.

### Generalizability and Interpretation

The satisfaction with the course was high in both groups, and participants found both educational interventions to be sufficiently interactive. It has been shown that participants’ satisfaction influences their academic scores [20] and learning motivation [21]. One of our concerns about the web-based training was the limitation of direct communication with the instructors. Participants might appreciate the ease of asking a question and getting an immediate reaction and answer in a face-to-face setting. However, newer technologies and new generations of students have superseded those concerns. It has been reported that web-based students ask more complex questions and more time is assigned by teachers for answering them [22].

There were no differences between the groups for the overall test results and SoF table understanding. Although median scores were low for both, they were lower for the 5 questions on methodology. These questions might be too advanced for the third-year medical students. Some of the individual questions had better response rates. The participants in the face-to-face group better answered the question on assessing the overall certainty of the evidence, taking into account the risk of bias, as well as other domains. Previous research in risk-of-bias education involved doctoral students [23], in which students who had a more intense training, including active discussions and feedback from lecturers, had better results. It is likely that our results stem from a similar involvement of participants and the lecturer, who could clarify certain aspects of evidence assessment students might have struggled with.

Both groups had high scores in the understanding of the SoF table, which is consistent with previous research on understanding evidence presented in this way [4,24]. The face-to-face training group was better at recognizing the reasons for assessing the evidence as very low, which is a question of accessibility and the ease of use of SoF tables. It is not known if the current SoF table format influences these outcomes [25], and our sample size limits the generalizability of such conclusions.

Clear and understandable communication of evidence that is important for clinical practice is a previously recognized priority [26]. Even though methodological guides for clinical practice guidelines and systematic reviews have instructions on how to write conclusions and recommendations [16,27,28], there are few, if any, assessments of the effectiveness of those instructions. One study found that using words related to activity and behavior, along with simple language and the avoidance of highly specialized terms and passive verbs, improved end users’ attitudes toward recommendations [29]. Our participants received no instructions on how to write a recommendation in this intervention, so the analysis of their recommendations using the NICE guideline instructions shows how an “instructions-naive” third-year medical students with limited clinical experience would write a recommendation.

Students often used scientific language and formed their answers as conclusions without elements of a clinical decision. Such distancing from recommending a clear action or against one might be explained as the result of students’ lack of clinical experience but also as a part of the culture of defensive medicine, in which medical professionals avoid a decisive action due to the fear of complications and responsibility [30]. It might also be a result of the culture of EBM, in which uncertainty and the need for more evidence are sometimes overemphasized [31]. Both of these elements are a part of the hidden medical curriculum that influences all students, and there might be a possibility of such a curriculum having a bigger impact in face-to-face education. Our findings are from a small sample size and warrant further research. Both groups of students mostly used patient-oriented language, with verbs such as “offer” instead of “give” and “prescribe.” Students should be taught to use more active, personal, and patient-oriented language in recommendations, because it gives patients a greater sense of control over their condition and behavior and improves their intention to use them [29].

### Limitations

This study included a sample of third-year medical students, with limited clinical experience. Clinical experience might alter the perception of outcome importance and the severity of unwanted effects, both of which can influence recommendations for clinical practice. This trial did not include clinically experienced medical students or other health care workers, and its results might not translate to such populations. Another possible limitation of this study is that it did not involve an official GRADE training. There are no official criteria or consensus for defining what constitutes a GRADE methodologist—someone who support the creation of a guideline or help systematic review authors in evidence assessment [32]. As there is no consensus for GRADE experts, it is not unexpected that there is no consensus in the required education for GRADE end users. As previous research showed that web-based learning is as effective as face-to-face learning in health training in general [33,34], and specifically for EBM teaching to medical students [35], we hypothesized there would be no significant differences between the 2 groups. Furthermore, our study used a single training session, which might seem insufficient for such a complex topic. However, the intervention was embedded in a regular EBM course and was focused on specific issues. We have used this approach in our previous research on EBM education [36,37] and demonstrated that even a single intervention can make a difference in outcomes, at least in short-term knowledge.

### Conclusions

EBM skills are necessary for decision-making in health care, but the transfer of this knowledge to practice is often inadequate [38]. The lack of knowledge, time, and access are some of the identified barriers to EBM application [39-41]. Our study has added to the body of evidence that shows the effectiveness of web-based EBM education [42-44] for undergraduate and postgraduate students [34,45]. The results of our study can be used as a starting point for future research on GRADE education as a part of EBM training for practicing physicians, perhaps in a clinically integrated manner, which was shown to be effective in improving EBM behavior as well as knowledge [46]. Preclinical medical students might benefit from this education as well. Even though they might lack nuances of clinical experience, early exposure to EBM principles and critical evidence assessment increases the likelihood of them using EBM principles in practice later on [38,47]. Our results provide encouraging data on the effectiveness and acceptability of a completely web-based, asynchronous educational content for that purpose.

## Appendix

Multimedia Appendix 1 Summary of Findings table and questions from the test.

Multimedia Appendix 2 CONSORT checklist.

## Abbreviations

CIL: Cochrane Interactive Learning
EBM: evidence-based medicine
GRADE: Grading of Recommendations Assessment, Development and Evaluation
NICE: National Institute for Health and Care Excellence
SoF: Summary of Findings

## Data Availability

The data sets generated during this study are available from the first author (RT) on reasonable request.
