# Supplementary material for: Similar Outcomes of Web-Based and Face-to-Face Training of the GRADE Approach for the Certainty of Evidence: Randomized Controlled Trial
Source: J Med Internet Res. 2023 Jun 6;25:e43928. doi: 10.2196/43928 (PMC10282904; doi:10.2196/43928)
Supplement: Multimedia Appendix 1 [file jmir_v25i1e43928_app1.docx]

**Tokalić et al. Similar outcomes of online and face to face training of GRADE approach for certainty of evidence: a randomized controlled study**

Supplement S1. Summary of Findings table used in the outcome assessment (Lazzerini M, Ronfani L. Oral zinc for treating diarrhoea in children. Cochrane Database Syst Rev. 2012 Jun 13;(6):CD005436.)

**SUMMARY OF FINDINGS FOR THE MAIN COMPARISON *[***[***Explanation***](http://www.thecochranelibrary.com/view/0/SummaryFindings.html)***]***

Oral zinc for treating diarrhoea in children (Review)

Copyright © 2017 The Authors. Cochrane Database of Systematic Reviews published by John Wiley & Sons, Ltd. on behalf of The Cochrane Collaboration.

| Zinc compared to placebo for children more than 6 months of age with acute diarrhoea | | | | | | | | | | | |
| --- | --- | --- | --- | --- | --- | --- | --- | --- | --- | --- | --- |
| **Patient or population:** children with acute diarrhoea  Settings: all countries, Intervention: zinc, Comparison: placebo | | | | | | | | | | | |
| Outcomes | Illustrative comparative risks* (95% CI) | | Relative effect (95% CI) | | Number of participants (trials) | Certainty of the evidence (GRADE) | | | Comments | | |
|  | Assumed risk | Corresponding risk |  |  |  |  |  |  |  |  |  |
|  | Placebo | Zinc |  |  |  |  |  |  |  |  |  |
| Duration of diarrhoea | All trials | | MD −11.46  (−19.72 to −3.19) | | 2581  (9 trials) | ⊕⊕  **low**1,2 |  |  | |  | No comment |
|  | The mean duration of diarrhoea among placebo ranged from **31.2 to 169.5 hours** | The mean duration of diarrhoea among zinc ranged from **28.8 to 147.6 hours** |  |  |  |  |  |  |  |  |  |
|  | Trials limited to children with signs of malnutrition | | MD −26.39  (−36.54 to −16.23) | | 419  (5 trials) | ⊕⊕⊕⊕  high |  |  | |  | No comment |
|  | The mean duration of diarrhoea among placebo ranged from **103.4 to 146.4 hours** | The mean duration of diarrhoea among zinc ranged from **70.4 to 120.0 hours** |  |  |  |  |  |  |  |  |  |
| Diarrhoea on day 7 | 128 per 1000 | 93 per 1000  (78 to 113) | RR 0.73  (0.61 to 0.88) | | 3865  (6 trials) | ⊕⊕⊕  moderate3 |  |  | |  | No comment |
| Number of children hospitalized (community trials only) | - | - | - | 276  (1 trial) | | ⊕  very low4,5 | | | No events | | |

Oral zinc for treating diarrhoea in children (Review)

Copyright © 2017 The Authors. Cochrane Database of Systematic Reviews published by John Wiley & Sons, Ltd. on behalf of The Cochrane Collaboration.

| Death | 5 per 1000 | 1 per 1000  (0 to 11) | RR 0.29  (0.04 to | 2.20) | 1134  (4 trials) | ⊕  very low6,7 | Few events |
| --- | --- | --- | --- | --- | --- | --- | --- |
| Adverse events (vomiting) | 119 per 1000 | 188 per 1000  (173 to 242) | RR 1.57  (1.32 to | 1.86) | 2605  (6 trials) | ⊕⊕⊕  moderate8 | No comment |
| * The basis for the **assumed risk** (for example, the median control group risk across studies) is provided in footnotes. The **corresponding risk** (and its 95% CI) is based on the assumed risk in the comparison group and the **relative effect** of the intervention (and its 95% CI).  Abbreviations: **CI:** confidence interval; **MD:** mean difference; **RR:** risk ratio. | | | | | | | |
| GRADE Working Group grades of evidence  **High certainty:** further research is very unlikely to change our confidence in the estimate of effect.  **Moderate certainty:** further research is likely to have an important impact on our confidence in the estimate of effect and may change the estimate.  **Low certainty:** further research is very likely to have an important impact on our confidence in the estimate of effect and is likely to change the estimate.  **Very low certainty:** we are very uncertain about the estimate. | | | | | | | |

1Downgraded by 1 for indirectness: all trials were conducted in Asia.

2Downgraded by 1 for serious imprecision: wide CI.

3Downgraded by 1 for serious indirectness: these trials were all conducted in Asia in countries at high risk of zinc deficiency.

4Downgraded by 1 for serious indirectness: only one small community trial reported on number of children hospitalized.

5Downgraded by 2 for very serious imprecision: no hospitalizations occurred in this trial.

6Downgraded by 1 for serious indirectness: the included trials were mostly conducted in hospitals and are therefore likely to underestimate death at the community level.

7 Downgraded by 2 for very serious imprecision: only three deaths occurred in these two trials, consequently the trials are

significantly underpowered to detect or exclude an effect.

8Downgraded by 1 for serious risk of bias: two trials reported no details on sequence generation, allocation concealment, blinding, and incomplete outcome data, while one did not give any details regarding allocation concealment.

**Demographic questions:**

1. Gender

2. Age in years

3. Level of completed education (high school, bachelor degree, master degree, other)

3. Authorship of a research publication in the last 5 years (yes/no)

4. Authorship of a clinical practice guideline (yes/no)

5. How familiar are you with Cochrane collaboration?

(scale from 1 – not at all to 5 – extremely familiar)

How would you grade your knowledge of GRADE approach? (median (95% CI), n=88)

(scale from 1 – very low to 5 – very high)

**Satisfaction with and opinion about the course** (on a scale from 1 – I do not agree at all to 7 – I fully agree)

1. Overall, I am satisfied with the course.
2. This course was really useful.
3. This is a good way for learning GRADE approach for quality of evidence.
4. This course helped me to better understand the concepts related to GRADE.
5. The course covered too much content in a short period of time.
6. I think there was sufficient amount of interaction during this course.
7. I would recommend this course to my colleagues.
8. I did not find this course useful.
9. In future, I will apply what I learned at this course in my work and research.
10. In future, I will learn more about interpreting and grading the quality of evidence.

**Questions related to the understanding the Summary of Findings table:**

1. Based on this information, how would you formulate a recommendation for clinical practice?

2. Would you consider any subgroups of patients, and if so, how?

3. How many participants were there in trials that assessed death as an outcome?

4. Why was the quality of evidence for hospitalized children graded as very low?

**Cochrane Interactive Learning (CIL) test (asterisks indicate correct answers):**

Now that you have completed the content for this module, you can test yourself in this formal assessment. This assessment consists of five questions.

**1. Understand confidence intervals in the interpretation of results of meta-analysis**


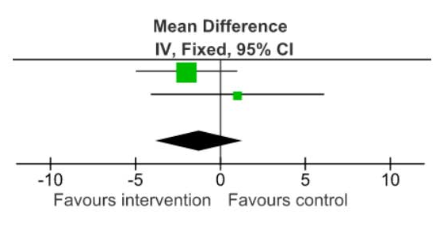
This forest plot is based on two studies including a total of 412 participants in the intervention group and 390 participants in the control group. Which of the following statements is correct?

**A.** Based on the confidence interval around the effect estimate, one can consider the intervention not to be effective

**B.** If the minimally important difference is two units, the intervention can be considered effective

**C.** If the minimally important difference is two units, the effect estimate can be considered imprecise*

**D.** One should describe the difference in effect as statistically non-significant

**2. Identify ways of re-expressing the standardized mean difference**

Which of the following statements regarding standardized mean difference (SMD) are correct? (more than one answer)

**A.** SMD is expressed in units of standard error

**B.** An odds ratio can be approximated from SMD*

**C.** The accuracy of re-expressing SMDs in the units of a familiar instruments depends on the range of mean differences in the included studies

**D.** Rules of thumb used to interpret SMDs can be explained in the Comments column of the ‘Summary of findings’ table*

**3. Interpret a funnel plot asymmetry**


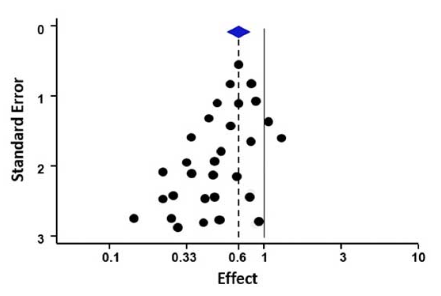
What can you conclude from this funnel plot? Select all that apply.

**A.** Smaller trials have results that are systematically different to larger trials*

**B.** The funnel plot asymmetry may be due to chance*

**C.** The funnel plot asymmetry may be due to clinical diversity*

**D.** Small studies that found no effect of intervention have not been published

**4. Determine the overall certainty of the evidence**

Authors reported that the risk ratio for the effect of vitamin D intake on the number of frail elderly people who have a hip fracture annually is 0.83 (95% CI 0.54 to 1.29). The risk of bias figure is provided below. What is the overall level of certainty of the evidence for hip fractures?

Choose all the correct answers.


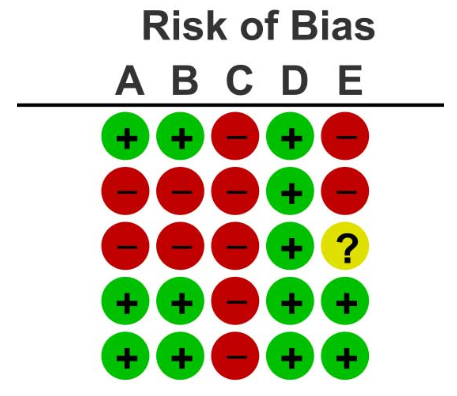


**A.** The risk of bias across studies is a serious concern, so I have moderate certainty in the effect I found

**B.** I can’t decide. I need more information about the number of people and events in the studies (i.e. about the imprecision of the results)*

**C.** I can’t decide. I need more information about whether the results are heterogeneous across studies (i.e. inconsistency)*

**D.** I can’t decide. I need more information about the types of people in the studies, and the dose of vitamin D provided (i.e. indirectness)*

**E.** I can’t decide. I need more information about whether it’s possible that important studies are missing (i.e. publication bias)*

**5. Decide on rating up a body of evidence**

Consider a review of randomized controlled trials for the effect of drug A compared to a placebo on the number of people cured. The authors assessed the evidence using GRADE and found that there was no concern with the risk of bias across the studies, there was no inconsistency or indirectness, and publication bias was undetected. Across the studies in the intervention and placebo group, there were only 120 people cured, so the evidence was rated down for imprecision. The effect was large though – a risk ratio of 3.4 with 95% confidence intervals from 2.1 to 4.7 – so the authors rated up the evidence. Therefore, the overall quality of evidence is high. Did the authors upgrade the evidence appropriately?

Choose the correct answer.

YES NO*
